# Serum NfL and EGFR/NfL ratio mRNAs as biomarkers for phenotype and disease severity of myelin oligodendrocyte glycoprotein IgG-associated disease

**DOI:** 10.3389/fimmu.2024.1388734

**Published:** 2024-05-14

**Authors:** Xin Wang, Yi Qu, Jiayu Fan, Huiqiang Ren

**Affiliations:** ^1^ Second Department of Neurology, Hebei Children’s Hospital, Shijiazhuang, China; ^2^ Department of Science and Education, Hebei Children’s Hospital, Shijiazhuang, China; ^3^ Department of Pathology, Hebei Children’s Hospital, Shijiazhuang, China

**Keywords:** myelin oligodendrocyte glycoprotein IgG-associated disease, biomarker, neurofilament light chain, endothelial growth factor receptor, acquired demyelinating syndrome

## Abstract

**Background and purpose:**

Myelin oligodendrocyte glycoprotein (MOG) IgG is frequently elevated in pediatric patients with acquired demyelinating syndrome (ADS). However, no specific biomarkers exist for phenotype classification, symptom severity, prognosis, and treatment guidance of MOG-IgG-associated disease (MOGAD). This study evaluated neurofilament light chain (NfL) and endothelial growth factor receptor (EGFR) mRNA expression levels in serum and cerebrospinal fluid (CSF) as potential biomarkers for MOGAD in Chinese children.

**Methods:**

This was a cross-sectional and single-center study. We enrolled 22 consecutive pediatric patients hospitalized with MOGAD and 20 control pediatric patients hospitalized for noninflammatory neurological diseases in Hebei Children’s Hospital. Serum and CSF were collected from MOGAD patients within 3 days before immunotherapy. The mRNA levels of NfL and EGFR in serum and CSF were measured by real-time polymerase chain reaction (qPCR), and the EGFR/NfL ratio mRNA was calculated. These measurement values were then compared between disease groups and among MOGAD phenotypes. In addition, the correlations between the mRNAs of three markers (NfL, EGFR, EGFR/NfL ratio), extended disability status scale (EDSS) scores, and clinical phenotypes were analyzed.

**Results:**

Serum and CSF NfL mRNA levels were significantly higher of acute-stage MOGAD patients than those of control patients (*p<* 0.05 and *p<* 0.01, respectively), while the mRNA levels of serum EGFR and EGFR/NfL ratio were significantly lower of MOGAD patients than those of controls (*p <* 0.05, *p <* 0.0001). Serum NfL mRNA was significantly correlated with mRNA of serum EGFR (*r* =0.480, *p* < 0.05). Serum and CSF NfL mRNA levels in MOGAD patients with the ADEM-like phenotype were also significantly higher than those in control patients (*p* < 0.01, *p* < 0.01) and optic neuritis (ON) phenotype (*p* < 0.05, *p* < 0.05). Both mRNAs of NfL in CSF and EGFR/NfL ratio in serum were correlated with EDSS scores (*p* < 0.05, *r* = 0.424; *p* < 0.05, *r*= -0.521).

**Conclusion:**

The mRNA levels of elevated NfL in serum and CSF as well as lower EGFR and EGFR/NfL ratio in serum could help distinguish acute-phase MOGAD. Higher mRNA levels of NfL in serum and CSF of MOGAD patients help distinguish ADEM-like phenotype. In addition, serum EGFR/NfL mRNA ratio is indicative of disease severity in pediatric patients with MOGAD. Further investigations are warranted to elucidate the pathological mechanisms underlying these associations.

## Introduction

Myelin oligodendrocyte glycoprotein (MOG) is an oligodendrocyte-specific biomolecule that is located in the outermost layer of the myelin sheath. It is believed to be essential for myelin stability, neuroimmune regulation, and various intracellular signaling functions (1). MOG-IgG-associated disorder (MOGAD) is a predominantly childhood-onset autoimmune demyelinating disease of the central nervous system (CNS) that may manifest with paraparesis, paralysis, vision and other sensory impairments, and seizures depending on the region affected (2–6). Although many patients with MOGAD are responsive to hormone therapy, some of them continue to experience relapse or residual effects. Predicting disease onset, symptom profile, relapse risk, and therapeutic response could be invaluable for clinical management. Nevertheless, no reliable biomarkers have been identified and tested for MOGAD.

Neurofilament light chain (NfL) is a component of the neuronal cytoskeleton that is released into the blood and CSF after axon damage (4, 7). Thus, serum NfL concentrations are elevated in neurodegenerative disorders and demyelinating diseases such as multiple sclerosis (MS) as well as following neurotrauma (8, 9). Moreover, NfL release is proportional to the extent of myelin damage in MS; hence, the serum or CSF concentration of NfL may be associated with disease severity, treatment efficacy, and long-term prognosis (10, 11). However, no studies have examined the relationships of serum and CSF NfL with disease parameters in children with MOGAD.

Epidermal growth factor receptor (EGFR) is a receptor tyrosine kinase that is often upregulated in different types of cancer. Recent studies have shown that EGFR expression may also be altered in various immunoinflammatory and autoimmune diseases (12). Further, EGFR can stimulate the maturation of oligodendrocytes after CNS injury and has metabolic effects on the mammalian spinal cord (13), suggesting potential associations with current clinical severity and outcome.

In the current study, we compared serum and CSF mRNA expressions of NfL and EFGR as well as the corresponding EGFR/NfL ratios between children with acute-stage MOGAD and children with non-demyelinating neurological diseases. This comparison was performed to assess the specificity of these values as diagnostic markers. In addition, we examined associations with specific MOGAD phenotypes and correlations with disease severity.

## Patients and methods

This was a cross-sectional and single-center study. We enrolled 22 pediatric inpatients diagnosed with MOGAD for the first acute episode in Hebei Children’s Hospital and collected serum and CSF samples within 3 days before immunotherapy. Serum and CSF samples were collected on an empty stomach in the morning. We also included as controls, blood and CSF samples from 20 age- and sex-matched children hospitalized at the same institution for non-demyelinating diseases, including febrile convulsions (*n* = 4), infectious meningitis (*n* = 6), and migraine (*n* = 10). Detailed diagnostic evaluations were conducted to exclude demyelinating diseases and demyelinating injuries.

The diagnosis of MOGAD was based on the criteria proposed by the International MOGAD Expert Group in 2023 (14). The patients included fulfilled the following criteria: i) pediatric patients were hospitalized in Hebei Children’s Hospital for the first episode of disease; ii) met diagnostic criteria of International MOGAD Expert Group in 2023; iii) serum MOG-IgG was positive, aquaporin-4 (AQP4)-IgG and other immune antibodies were negative, which was confirmed by live cell cell-based assays; iv) except congenital demyelinating syndrome and other genetic or metabolic diseases.

Two neurologists collected clinical data including age, gender, triggering events, days of hospitalization, symptoms, brain magnetic resonance imaging (MRI) findings, clinical phenotype, treatments, and expanded disability status scale (EDSS) scores, at the time of blood and CSF sampling. All CSF samples collected by lumbar puncture were acquired for diagnosis or treatment.

### Preparation of the samples

Serum and CSF samples were collected within 3 days before immunotherapy, on an empty stomach in the morning. The samples were centrifuged immediately after collected and stored at −80°C until assayed for NfL and EGFR mRNA levels.

### RNA extraction and quantitative real-time polymerase chain reaction

Real-time polymerase chain reaction (qPCR) was used to quantify the expression levels of NfL and EGFR mRNAs in serum and CSF. Briefly, total RNA was extracted using TRIzol solution according to the manufacturer’s instructions. RNA concentration and purity were determined using a NanoDrop^®^ ND-2000 (CW0623S, Jiangsu, China) spectrophotometer, while RNA integrity was determined by denatured agarose gel electrophoresis. Total RNA samples obtained were then reverse transcribed into cDNA using the HiScript III 1st Strand cDNA Synthesis Kit (+gDNA wiper) (R312-01, Nanjing, China) according to the manufacturer’s instructions. Quantitative real-time PCR was performed on a Rotor-Gene Q instrument (BIO-RAD, Shijiazhuang, China) using real-time Master Mix SYBER Green (CW0957, Jiangsu, China) and the primers listed in **Table 1**. Gene expression was calculated using the ΔΔCt method and normalized to controls.

**Table 1:** The primers information of genes in this study.

Gene		Primers sequence (5’to3’)	Amplicon length (bp)
EGFR	Forward	CGCTACCTTGTCATTCAG	101
EGFR	Reverse	ACGTCGTCCATGTCTTCT	
NfL	Forward	CAGCGTGGGAAGCATAAC	78
NfL	Reverse	GTCTGTAAACCGCCGTAG	
GAPDH	Forward	CACCCACTCCTCCACCTTTGA	188
GAPDH	Reverse	TCTCTCTTCCTCTTGTGCTCTTGC	

EGFR, Epidermal growth factor receptor. NfL, neurofilament light chain; GAPDH, glyceraldehyde 3-phosphate dehydrogenase.

### Statistical analysis

All statistical analyses were conducted using Statistical Package for Social Sciences (SPSS) 23.0. Depending on whether the dataset has normality and homogeneity of variance, results are expressed as mean ± standard deviation (SD) or median (interquartile range, [IQR]). Two normally distributed datasets were compared by independent samples t-test, while two non-normally distributed data sets were compared Mann-Whitney U test. More than two normally distributed datasets were compared by ANOVA and Scheffe correction for pairwise comparison. Associations between factors were evaluated by calculating Spearman test correlation coefficients. A value of *p* < 0.05 (two-tailed) was considered to be statistically significant for all tests.

## Results

### Demographic and clinical characteristics of MOGAD and control patient groups

A total of 22 children with MOGAD (12 females and 10 males, 6.96 ± 2.98 years) were recruited. In 14 cases (14/22, 63.6%), disease onset was associated with a precursor event (infection or vaccination) while no such event was identified in the remaining 8 cases (8/22, 36.4%). The main clinical manifestations were drowsiness (12/22,54.5%), fever (10/22,45.5%), convulsion (6/22,27.3%), movement disorder (5/22,22.7%) and vision loss (3/22,13.6%). The brain MRI lesions mainly involved subcortical white matter (16/22,72.7%), thalamus/basal ganglia area (14/22,63.6%), cerebellum (10/22,45.5%), brain stem (8/22,36.4%), the corpus callosum (6/22,27.3%) and optic nerve (3/22,13.6%). Most patients had multiple lesions (19/22,86.4%). Six patients (6/22, 27.3%) brain MRI lesions completely disappeared, and 16 (16/22,72.7%) improved. Four (4/22,18.2%) showed linear meningeal enhancement, which is more common in non-ADEM encephalitis pediatric patients. The median number of days of hospitalization was 24.0 [18.8–25.0] and the median EDSS score was 1.5 [1.5–2.0]. Nineteen patients (19/22, 86.4%) received first-line immunotherapy with intravenous methylprednisolone sodium succinate (20mg/kg·d,3-5d) and/or immunoglobulin (1g/kg·d, 2d). Three (3/22, 13.6%) received escalation therapy, 2 patients receiving rituximab (375mg/m^2^, q1w) and 1 patient receiving tocilizumab (12mg/kg·d, q4w). Neither serum NfL, CSF NfL, serum EGFR, nor CSF EGFR differed by age at sampling, sex, preceding event (or not), days of hospitalization, or treatment did not differ significantly from the control group (**Table 2**).

**Table 2:** Comparison of basal characteristics and biomarkers in pediatric MOGAD.

Basal characteristics (*n*=22)	mean ± SD or median (IQR) or *n* (%)	serum-NfL	CSF-NfL	serum-EGFR	CSF-EGFR
Age(years)	6.96 ± 2.98	*p=*0.479 *r*=0.014	*p=*0.329 *r*=0.099	*p=*0.254 *r*=0.167	*p=*0.387 *r*=0.065
Gender *n* (%)					
Male Female	10 (45.5) 12 (54.5)	*p=*0.697	*p =*0.652	*p=*0.829	*p=*0.797
Preceding event *n* (%)		*p=*0.285	*p=*0.365	*p=*0.930	*p=*0.764
Respiratory infection	14 (63.6)				
none	8 (36.4)				
Days of hospitalization, median (IQR)	24.0 (18.8-25.0)	*p=*0.278 *r*=0.270	*p=*0.295 *r*=0.234	*p=*0.363 *r*=0.228	*p=*0.155 *r*=0.314
clinical phenotype *n* (%)					
Encephalitis&ADEM	8 (36.4) & 8 (36.4)	*p=*0.126	*p=*0.010	*p=*0.990	*p=*0.452
encephalitis&ON	8 (36.4) & 3 (13.6)	*p=*0.905	*p=*0.194	*p=*0.413	*p=*0.085
encephalitis &myelitis	8 (36.4) & 3 (13.6)	*p=*0.393	*p=*0.990	*p=*0.990	*p=*0.921
ADEM&ON	8 (36.4) & 3 (13.6)	*p=*0.019	*p=*0.024	*p=*0.914	*p=*0.049
ADEM&myelitis	8 (36.4) & 3 (13.6)	*p=*0.292	*p=*0.497	*p=*0.990	*p=*0.630
myelitis&ON	3 (13.6) & 3 (13.6)	*p=*0.990	*p=*0.700	*p=*0.990	*p=*0.200
Treatment *n* (%)		*p=*0.824	*p=*0.164	*p=*0.586	*p=*0.464
First-line treatment	19 (86.4)				
Escalation treatment	3 (13.6)				
EDSS score at sampling, median (IQR)	1.5 (1.5-2.0)	*p=*0.652 *r*=0.102	*p=*0.039 *r*=0.424	*p=*0.801 *r*=0.064	*p=*0.085 *r*=0.303

ADEM, acute disseminated encephalomyelitis; CSF, cerebrospinal fluid; EDSS, Expanded Disability Status Scale; ON, optic neuritis.

### Elevated NfL mRNA expression in serum and CSF of pediatric MOGAD patients compared to controls

**Figure 1:**
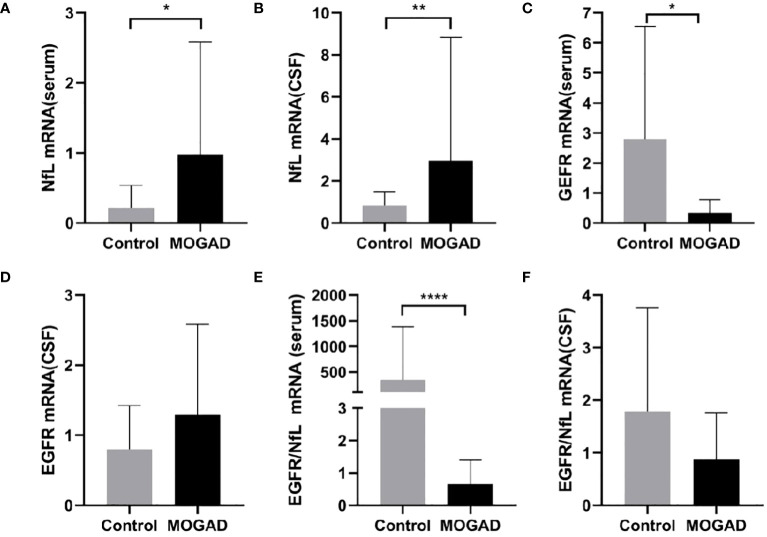
The mRNA levels of NfL in serum **(A)** and CSF **(B)**, EGFR in serum **(C)** and CSF **(D)**, EGFR/NfL ratio in serum **(E)** and CSF **(F)** between MOGAD and controls. (*p < 0.05, ** *p* < 0.01, **** *p* < 0.0001). MOGAD, Myelin oligodendrocyte glycoprotein IgG-associated disease; CSF, cerebrospinal fluid; NfL, neurofilament light chain; EGFR, endothelial growth factor receptor.

Serum NfL mRNA expression was significantly higher in MOGAD patients than control patients with noninflammatory neurological diseases (0.98 ± 1.60 vs. 0.21 ± 0.32, *p* = 0.031, *p* < 0.05) (**Figure 1A**). Similarly, NfL mRNA in CSF was higher in MOGAD patients than controls (2.97 ± 5.87 vs. 0.84 ± 0.66, *p* = 0.007, *p* < 0.01) (**Figure 1B**).

### Lower EGFR mRNA expression in serum of pediatric MOGAD patients compared to controls

Serum EGFR mRNA expression was significantly lower in the MOGAD group than the control group (0.34 ± 0.45 vs. 2.80 ± 3.74, *p* = 0.022, *p* < 0.05) (**Figure 1C**). However, there was no significant difference in CSF EGFR mRNA expression between MOGAD and control patients (0.80 ± 0.62 vs. 1.29 ± 0.29, *p* = 0.204) (**Figure 1D**).

### Lower EGFR/NfL mRNA ratio in the serum of pediatric MOGAD patients compared to controls

The serum EGFR/NfL ratio mRNA expression was significantly lower in MOGAD patients than controls (0.31 [0.04–1.27] vs. 3.51 [1.85–107.3], *p* < 0.0001) (**Figure 1E**). In contrast, the EGFR/NfL ratio mRNA expression in CSF did not differ between MOGAD patients and controls (0.56 [0.29–1.05] vs.1.04 [0.29–2.24], *p* = 0.299) (**Figure 1F**).

### Correlations of measured factors with pediatric MOGAD severity

**Figure 2:**
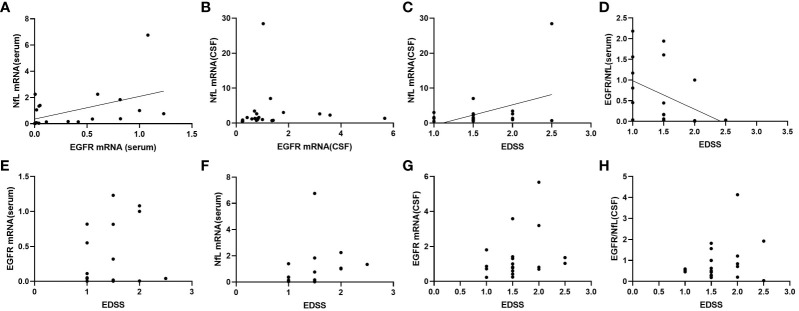
Correlations between mRNA expression of NfL and EGFR in serum **(A)** and CSF **(B)**, and correlations between mRNA expression of NfL in CSF **(C)**, EGFR/NfL ratio in serum **(D)**, EGFR in serum **(E)**, NfL in serum **(F)**, EGFR in CSF **(G)**, EGFR/NfL ratio in CSF **(H)** and EDSS in patients with MOGAD. CSF, cerebrospinal fluid; NfL, neurofilament light chain; EGFR, endothelial growth factor receptor; EDSS, expanded disability status scale.

Serum NfL mRNA expression was correlated with EGFR mRNA expression (*r* =0.480, *p* < 0.05), while the CSF levels of these factors were not correlated (**Figures 2A, B**). In addition, both mRNA levels of CSF NfL and serum GFER/NfL ratio were correlated with EDSS (*r*= 0.424, *p* < 0.05; *r*= -0.521, *p* < 0.05) (**Figures 2C, D**). There were no significant correlations between EDSS and serum EGFR (*p* = 0.801), serum NfL (*p* = 0.652), CSF EGFR (*p* = 0.085), and CSF EGFR/NfL ratio (*p* = 0.441) (**Figures 2E–H**).

### Differences in NfL and EGFR mRNA levels among MOGAD clinical phenotypes

**Figure 3:**
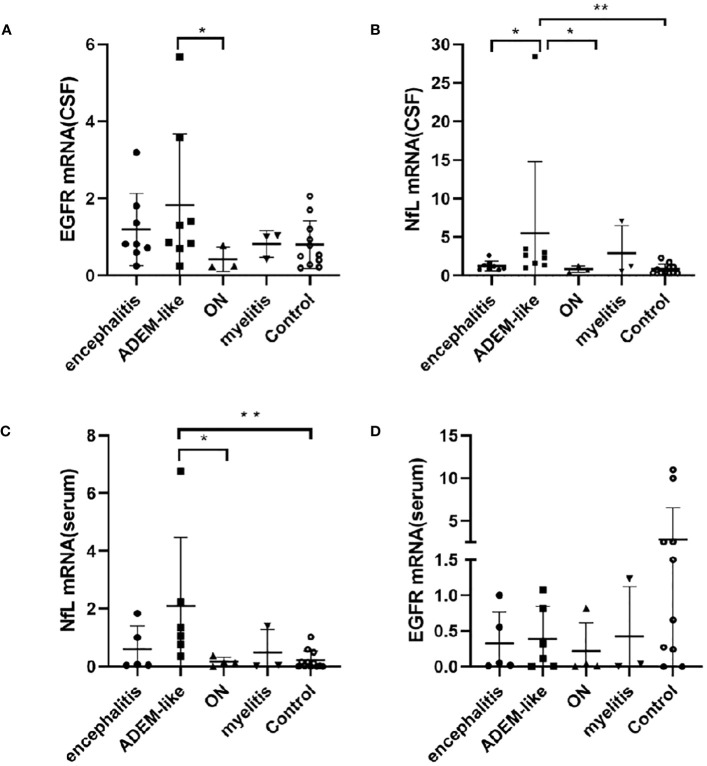
Correlations between mRNAs levels of EGFR in CSF **(A)**, NfL in CSF **(B)**, NfL in serum **(C)**, EGFR in serum **(D)** and phenotypes in patients with MOGAD. (*p < 0.05, ** p < 0.01). ADEM-like, acute disseminated encephalomyelitis-like, encephalitis (non-ADEM-like); ON, optic neuritis; CSF, cerebrospinal fluid; NfL, neurofilament light chain; EGFR, endothelial growth factor receptor.

The common phenotypes of MOGAD patients included acute disseminated encephalomyelitis (ADEM)-like, encephalitis (non-ADEM-like), optic neuritis (ON), and myelitis. The CSF EGFR mRNA level was significantly higher in the ADEM-like phenotype than the ON phenotype (*p* = 0.049, *p* < 0.05) (**Figure 3A**), and the CSF NfL mRNA level was significantly higher in the ADEM-like phenotype than that in the encephalitis phenotype (*p* = 0.010, *p* < 0.05), ON phenotype (*p* = 0.024, *p* < 0.05), and control non-myelinating diseases (*p* = 0.002, *p* < 0.01) (**Figure 3B**). In addition, the serum NfL mRNA level was higher in the ADEM-like phenotype than that in ON phenotype (*p* = 0.019, *p* < 0.05) and non-myelinating diseases (*p* = 0.0013, *p* < 0.01) (**Figure 3C**). Serum EGFR mRNA expression did not differ significantly among clinical phenotypes (**Figure 3D**).

## Discussion

Reliable biomarkers are urgently needed to improve the accuracy of pediatric ADS diagnosis and prognosis. NfL is a neuron-specific protein that maintains the structural stability of the axon cytoskeleton. Under normal conditions, low levels of NfL are constantly released from axons, probably in an age-dependent manner. However, in response to axonal damage (15), nutrient loss, oligodendrocyte damage, secondary degeneration, mitochondrial damage, and axonal energy failure (16), the release of NfL sharply increased, which is released into the CSF, and then drained into the blood (17). To date, however, studies on the associations of serum or CSF NfL content with MOGAD in children are lacking. Our study found significantly higher levels of NfL mRNA levels which were actually measured in both CSF and serum samples from children of untreated acute-onset MOGAD compared with children having non-demyelinating neurological disorders, suggesting that MOGAD onset may be associated with acute axonal and myelin injury or secondary pathological processes. Elevated NfL mRNA levels which were actually measured in CSF were also associated with disease severity as assessed by the EDSS, suggesting that higher levels reflect more widespread damage to axonal structures. We suggest that serum NfL might be a valuable and accessible biomarker to assist in MOGAD diagnosis.

Numerous studies have demonstrated the value of serum NfL for assessing the current severity of MS pathology (18–20). For instance, higher serum NfL levels are predictive of a faster increase in lesion volume on MRI (21) and are correlated with the number of new MRI lesions (22). Associations with CSF NfL are also presumed but not widely reported as sampling is highly invasive. Our study found that serum and CSF NfL levels were higher in MOGAD patients with the ADEM-like phenotype than the ON phenotype and higher than in patients with nonmyelinating disease. Children with the ADEM-like phenotype of MOGAD usually have large, blurry, bilateral, and extensive lesions on MRI that primarily affect the white matter and subcortical areas (18–20, 23). The differences in serum and CSF NfL among phenotypes may be related to the larger size of brain MRI lesions in the ADEM-like phenotype than the ON phenotype due to more severe demyelination and axonal injury. Elevated NfL levels in serum and CSF may also be the result of white matter damage from persistent neuroinflammation. Among pediatric ADS patients, those with ADEM exhibited the most extensive impairments in brain growth after a single demyelinating event (24). These associations may be helpful for the early identification of the ADEM phenotype for timely individualized treatment.

Epidermal growth factor receptor (EGFR) is a multifunctional transmembrane glycoprotein essential for proper neuron, astrocyte and oligodendrocyte development, neural circuit formation, axon compensation, neurotransmission, and synaptic plasticity (25, 26). The unidirectional penetration of endogenous EGF into the CNS parenchyma through the blood-brain barrier has been reported (27). The major components of the EGF-EGFR system in mammalian adult CNS and the transport of blood and CNS EGF have been identified (28). By promoting oligodendrocyte and axonal development, EGFR signaling is essential for specific stages of white matter formation (29). However, EGFR hyperphosphorylation, astrocyte activation, and proinflammatory cytokine production lead to demyelination, glial scarring, and oligodendrocyte destruction (30). When neuroinflammatory pathways are activated, ensuing changes in EGFR signaling affects oligodendrocyte maturation and inhibits myelin regeneration around damaged neurons (31). Surprisingly, EGFR inhibitors can promote axon regeneration, reduce myelin loss, promote the upregulation of growth-related proteins, and ultimately improve the recovery of limb motor function after spinal cord injury (32). This relation suggests that EGFR plays a dual role in controlling oligodendrogenesis and myelin regeneration depending on the activation of other signaling pathways (33). We found that serum EGFR level was significantly lower in MAGOD patients than age-matched nonmyelinating disease patients, while CSF EGFR level did not differ significantly between these clinical groups. After CNS injury, serum EGFR can enter the CNS through the blood–brain barrier, where it promotes the nutritive effects of cobalamin (vitamin B12) on myelin in oligodendrocytes and oligodendrocyte progenitors, as well as the multidirectional differentiation and proliferation of astrocytes (34). The difference in EGFR expression between serum and CSF may be related to distinct mechanism of action at these sites. Therefore, the associations of serum and CSF EGFR levels with disease status are likely complex and context dependent. Nonetheless, changes in serum EGFR may be useful for the differential diagnosis of MAGOD when combined with other clinical biomarkers and symptom patterns.

Serum EGFR level was significantly associated with serum NfL level, suggesting that the maturation of oligodendrocytes and subsequent myelination in MOGAD require both EGFR and NfL signaling (as well as other unknown signaling pathways). As EGFR can enhance the density and maturation of myelin-expressing oligodendrocytes and promote myelin regeneration after injury (35), EGFR signaling is a potential therapeutic target for enhancing axon regeneration after CNS injury despite the development of a myelin-inhibiting microenvironment (36). The molecular signaling pathways linking EGFR, NfL, and related inflammatory factors, and the specific contributions of these factors to MAGOD pathogenesis warrant further study to identify effective therapeutic targets.

In accordance with evidence that higher serum NfL levels reflect lesion size in MS (18–22), a few studies have found that serum NfL levels are associated with sustained axonal injury and significantly correlated with MS symptom severity and progression (37, 38). Also, serum NfL levels predicted the long-term disability course of MS and were strongly associated with higher EDSS and prolonged disease duration (39); moreover, serum NfL decreased after immunotherapy (40). Therefore, serum NfL could be useful for the early identification of high-risk MS patients (41). In adult patients with MOGAD, serum NfL levels were associated with seizure severity and disease activity (42, 43). However, few studies have been conducted on CSF NfL levels in children with MOGAD. The main therapeutic goals of MOGAD treatment are to suppress inflammation, reduce axonal damage and demyelination, and improve quality of life. Our study found that EDSS was significantly associated with NfL in CSF but not in serum, a finding at odds with previous studies. This discrepancy may be due to the significantly higher and thus more accurately measurable NfL concentrations in CSF than in serum or the heterogeneity of the selected cases. Therefore, these changes in serum and CSF NfL levels require further study in larger samples. We also found that the serum EGFR/NfL ratio was significantly correlated with EDSS. The utility of NfL alone as a biomarker may be limited as it is released by brain injury independent of etiology. Compared to CSF collection, serum is stable and easy to obtain. Therefore, the serum EGFR/NfL ratio may be a particularly valuable biomarker to evaluate the severity, progression, and treatment response of pediatric MOGAD patients. Frequent serum EGFR/NfL ratio measures may allow for the precise monitoring of disease activity, the timely identification of critical patients, and better treatment decisions, such as the timing of disease-modifying therapy upgrade.

Taken together, our study suggests that changes in serum and CSF NfL levels may be useful biomarkers for the early identification of acute-onset MAGOD and the differential diagnosis of clinical phenotypes. In addition, the serum EGFR/NfL ratio may be especially valuable as a biomarker of disease activity and severity in pediatric MOGAD patients. Further studies are needed to identify the cytokine pathways linking NfL and EGFR in MOGAD and the associations with the underlying pathological processes.

This study has several limitations. The small sample resulted in the under- and overrepresentation of certain phenotypes (such as the high proportion of ADEM patients). And, we cannot exclude that some patients may have suffered from systemic infection with potential CNS involvement, which may also increase blood NfL levels (44). Further, disease status was measured using only the EDSS as there are no other standardized assessment tools. Nonetheless, further research should also include tools to assess quality of life indicators and cognitive function.

## Data availability statement

The raw data supporting the conclusions of this article will be made available by the authors, without undue reservation.

## Ethics statement

The studies involving humans were approved by Medical Research Ethics Committee of Hebei Children’s Hospital. The studies were conducted in accordance with the local legislation and institutional requirements. Written informed consent for participation in this study was provided by the participants’ legal guardians/next of kin.

## Author contributions

XW: Writing – original draft, Writing – review & editing. YQ: Conceptualization, Writing – original draft. JF: Investigation, Writing – original draft. HR: Methodology, Writing – original draft.
